# Using developmental regression to reorganize the clinical importance of autistic atypicalities

**DOI:** 10.1038/s41398-022-02263-8

**Published:** 2022-12-01

**Authors:** David Gagnon, Abderrahim Zeribi, Élise Douard, Valérie Courchesne, Guillaume Huguet, Sébastien Jacquemont, Mor Absa Loum, Laurent Mottron

**Affiliations:** 1 Research Center of the CIUSSS-NIM, Hôpital Rivière-des-Prairies, 7070 boul. Perras, Montréal, QC H1E 1A4 Canada; 2 Department of Psychiatry and Addictology, University of Montreal, 2900, boul. Édouard-Montpetit, Montreal, QC H3T 1J4 Canada; 3 Department of Pediatrics, University of Montreal, 2900, boul. Édouard-Montpetit, Montreal, QC H3T 1J4 Canada; 4 Sainte-Justine Research Center, 3175, chemin de la Côte-Sainte-Catherine, Montreal, QC H3T 1C5 Canada; 5 Psychoeducation School, University of Montreal, C.P. 6128, Succ. Centre-Ville, Montréal, QC H3C 3J7 Canada; 6 Research Center of the CIUSSS-EMTL, 7331, rue Hochelaga, Montréal, QC H1N 3V2 Canada; 7 Campbell Family Mental Health Research Institute (CAMH), 1001 Queen Street West, Toronto, M6J 1H4 ON Canada

**Keywords:** Autism spectrum disorders, Diagnostic markers

## Abstract

Early regression (ER) is often reported in autistic children with a prototypical phenotype and has been proposed as a possible pathognomonic sign present in most autistic children. Despite the uncertainties attached to its definition and report, using ER to anchor the autism phenotype could help identify the signs that best contribute to an autism diagnosis. We extracted retrospective data from 1547 autistic children between the ages of 6 and 18 years from the Simons Simplex collection. Logistic regression identified the atypicalities associated with a history of ER. Stepwise variable selection using logistic regression analysis followed by a bootstrap procedure of 1000 iterations identified the cluster of atypicalities best associated with ER. Linear and logistic regressions measured the association between combinations of atypicalities within the identified cluster and adaptative behaviors, diagnostic areas of severity, and other categories. Seven atypicalities significantly increased the likelihood of having experienced ER (OR = 1.73–2.13). Four (“hand leading—ever”, “pronominal reversal—ever”, “never shakes head at age 4–5” and “stereotypic use of objects or interest in parts of objects—ever”), when grouped together, best characterized the phenotype of verbal autistic children with ER. This clustering of signs was associated with certain persistent language difficulties, higher summary scores on a diagnostic scale for autism, and greater odds of receiving an “autistic disorder” diagnosis instead of another pervasive developmental disorder (PDD) diagnosis. These results raise questions about using language as a clinical specifier, defining cross-sectional signs independent of their relationship with an early developmental trajectory, and relying on polythetic criteria or equivalent weighted autistic atypicalities.

## Introduction

The reported increase in the prevalence and heterogeneity of autism challenges its nosology, as well as its semiology, i.e., the organization of signs and symptoms that ground clinical reasoning. For the most recent classifications of autism, autistic signs are organized into two domains: deficits in social communication and social interaction and restricted, repetitive patterns of behavior, interests, or activities [1]. An autism diagnosis in clinical and research settings is most commonly established using a combination of beyond-threshold scores in retrospective (ADI-R) and observational (ADOS) instruments in addition to clinical judgment. In the diagnostic algorithms of these instruments, each sign with diagnostic value contributes equally to reaching, or not reaching, a within-domain threshold in diagnostic areas, which varies according to the instrument.

Current diagnostic practices result in heterogeneity, which has a major impact on research, clinical care, and the delivery of assistance, which is negative for some [2–4] but positive for others [5]. Researchers have tried to unravel subgroups based on clinical signs identified by standardized tools. According to a review of subgrouping strategies and results [6], the results are disappointing, unvalidated, nonreproducible, and arbitrarily selected. Critically, the subgroups thus obtained depend mostly on symptom scores integrated into a polythetic diagnostic system. Polythetic criteria are a way to obtain a diagnosis when the summary score of identified signs or symptoms reaches the diagnostic threshold, regardless of their respective weight and their possible combinations. Using polythetic criteria allows a large number of combinations of signs to reach the required threshold and does not take into account their potential unequal weight, distinctiveness, or specificity.

An alternative way to determine a possible group of atypicalities/signs of higher diagnostic relevance would be to determine clinical atypicalities that cluster with a cardinal sign that is sufficiently specific and sensitive. An early break in the development of social-communication skills, such as early regression (ER), appears to be a good starting point. Previously considered as a subgroup in studies based on retrospective assessments [7–11], the regressive/plateau phenotype is present, to various degrees, in a majority of autistic children when evaluated prospectively [12] and in ~32% when assessed retrospectively [13]. ER is more frequent among children who receive a diagnosis of the autistic disorder instead of other DSM-IV pervasive developmental disorders (PDD) [8, 14, 15]. It is not often reported by caregivers in other conditions [8, 10, 16], and it may represent a “signature” of an early autism developmental trajectory [17]. Here, we identified the main atypicalities that aggregate with reported ER. We then tested the validity of the selection of atypicalities by assessing the effect of their aggregation with the intensity of the autistic phenotype according to a standardized tool score and clinical assessment.

## Methods

### Participants

A sample of 1547 unrelated autistic individuals, aged 6–18 years, for whom all applicable retrospective items from the three areas of the ADI-R (#29 to #79 inclusive) are documented, were drawn from the Simons Simplex Collection (SSC). Only individuals aged six years or older were included for the retrospective ADI-R questions specific to the period of 4–5 years of age to be valid. Participants were diagnosed with DSM-IV autism, PDD-NOS, or Asperger’s disorder based on the clinicians’ best judgment. All underwent administration of the ADOS [18] and ADI-R [19] by experienced raters. Individuals had no other neurodevelopmental diagnoses at the time of enrollment and a mental age over 18 months (see www.sfari.org). This study was approved by the Research Ethics Board of the Centre Hospitalier Universitaire Sainte-Justine. Informed consent was obtained from all participants included in the Simons Simplex Collection at the time of their initial enrollment.

### Measures

The *ADI-R* retrospectively documents early developmental milestones, as well as the emergence or presence of autistic signs in the three diagnostic domains. Forty-nine retrospective ADI-R items (#29 to #79 inclusively) address the atypicalities of the three diagnostic areas. ADI-R items #30 (“general current level of language”) and #65 (“friendship”) were excluded from the analyses, as they are not retrospective measurements. The definition of ER used includes a history of a loss of five or more words, documented by item #11, and/or a loss of other skills, documented by item #20, or both for at least three months. Communicative items #32 to #41 were extracted only for verbal individuals. Most of the ADI-R items are scored using an ordinal severity scale from 0 to a maximum of 4, which was dichotomized to facilitate analysis: a score ≤1 indicated the absence of an atypicality, and a score ≥2 indicated the unequivocal manifestation of atypicality. Total ADI-R sum scores for the reciprocal social interaction and restricted and repetitive domains were used as a proxy of the stringency of the autistic phenotype for these areas.

The *Autism Diagnostic Observation Schedule-Generic (ADOS*) is a semi-structured observational assessment administered by trained clinicians. The child’s level of spoken language determines the choice of ADOS modules. Modules 3 and 4 assess individuals with “fluent speech”, defined as the “spontaneous, flexible use of sentences with multiple clauses that describe logical connections within a sentence” [18]. Module 3 was chosen as the cutoff to avoid ambiguity about the child’s verbal level at enrollment, thereby preventing any inconsistency between the language level reported on the ADI-R and determined by the choice of ADOS module. The ADOS-calibrated severity scores were used as a proxy for the severity of the autistic areas at the time of enrollment [20, 21].

The *Vineland-Second Edition* (VABS) [22] is a standardized, semi-structured interview with the caregiver that assesses adaptive abilities, including expressive and receptive communication subdomains; the results were used as an additional measure of communication level.

*The Peabody* Picture Vocabulary 4th edition (PPVT-4) [23] is a direct, standardized assessment of vocabulary knowledge through visual image recognition and was used as a measure of receptive language.

The standard score from the *nonword repetition subtest* (NWR) of the Comprehensive Test of Phonological Processing [24] primarily measures phonological memory.

*Verbal IQ* and *nonverbal IQ* scores were obtained from the Differential Ability Scales-Second Edition Early Years/School Age [25], Mullen Scales of Early Learning [26], Wechsler Abbreviated Scale of Intelligence First Edition [27], and Wechsler Intelligence Scale for Children 4th Edition [28].

The clinical diagnostic impression (according to DSM-IV nosology), according to the “best judgment of the clinician”, was extracted from the variable “non-standardized impressions” of the diagnostic information of the SSC. This variable was then dichotomized according to whether the selected diagnosis was “autistic disorder”.

### Analyses

Logistic regression analyses, which were Bonferroni corrected, estimated the association of each atypicality with a history of ER; these analyses were performed on the entire sample and then only for verbal individuals. A bidirectional stepwise variable selection procedure on ADI-R items, based on the Bayesian Information Criteria (BIC) of logistic regression, was performed on the sample of verbal individuals to identify the combination of atypicalities (including all communication atypicalities only documented in the sample of verbal individuals) that best describe the phenotype of autistic individuals who have experienced ER. Variable selection in the stepwise analyses was directed by the BIC criterion, which is a penalized likelihood model selection criterion used to compare different models. We used the BIC criterion with the stepwise approach because it favors a model that includes the fewest variables. As a result, it creates a final model with the minimum number of atypicalities that best predicts ER. BIC criteria rather than Akaike information criteria (AIC) were used to reduce the risk of false positives inherent to stepwise analysis [29]. The same analysis was finally performed with 1000 samples in a bootstrap procedure using the “boot.stepAIC()” function of R statistical software [30] to evaluate the consistency of the stepwise variable selection in samples created by a random replacement procedure. Bootstrap analysis is a well-known validation approach [6]. We chose 1000 samples as a reasonable limit, as it has already been used by us [31–33] and others [34–36]. All of the above models were adjusted for nonverbal IQ, sex, and age at the time of assessment to control for possible memory bias. The phi coefficient between the ADI-R dichotomized items was calculated prior to the stepwise procedure to ensure the independence of items. None were >0.5, with the exception of “Never shakes head at age 4–5 years” and “Never nods head at age 4 to 5 years”, which had a phi coefficient of 0.75 (Supplementary Fig. S1). The statistical analyses were performed using R software version 4.1.3 [37] (see Appendix 1 for the specific packages).

A categorical variable representing the number of atypicalities shown for each verbal individual (one, two, three, etc.) among those selected by the stepwise analysis was created. Logistic regression analyses estimated the odds of receiving a clinical diagnosis of “autistic disorder” according to this variable, i.e., the number/combinations of atypicalities. The analysis was adjusted for nonverbal IQ, sex, and age at the time of assessment. Linear regression was used to estimate the effect of the combination of selected atypicalities on language/communication abilities and the stringency of the autistic phenotype. The dependent variables underwent, if necessary, the appropriate transformations required by a linear regression model. All were normalized to a z score, using the mean and standard deviation from the sample, for comparative purposes. Each linear and logistic regression was adjusted for sex, nonverbal IQ, age at the time of assessment and met the required statistical assumptions.

## Results

**Fig. 1:**
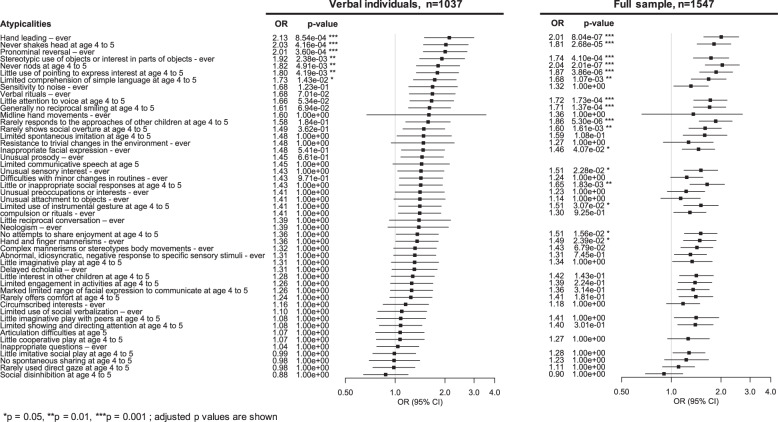
Association between autistic atypicalities and ER. Odds ratios (OR) and 95% confidence intervals are shown. Significance level is 0.05 after Bonferroni correction; adjusted p values are shown.

The sociodemographic information of the participants is presented in Table 1. In our sample of participants aged 6 years or older, 23% of verbal individuals and 32% of all individuals had previously experienced ER. The set of atypicalities documented for each participant are listed in Fig. 1 and Supplementary Fig. S1.

**Table 1:** Demographic and cognitive data of participants with and without ER.

	Verbal participants, *n* = 1037		Full sample, *n* = 1547	
	ER	No-ER	ER	No-ER
*n* (%)	242 (23)	795 (77)	496 (32)	1051 (68)
Mean age in months (SD)	131 (41)	124 (37)	124 (40)	122 (37)
Sex (male), %	90%	89%	88%	87%
Ethnicity (Caucasian vs. other), %	81%	84%	74%	80%
Mean IQ				
Verbal IQ (SD)	90 (20)	95 (22)	65 (33)	84 (30)
Nonverbal IQ (SD)	92 (19)	96 (19)	75 (28)	88 (25)

### Autistic atypicalities associated with early regression

Seven atypicalities significantly increased (by 1.73–2.13 times) the odds of having previously experienced ER among verbal individuals (Fig. 1 and Supplementary Table S1): “hand leading—ever”, OR = 2.13, 95% CI [1.5–3.0], *P* = 8.5e-4; “never shakes head at age 4 to 5”, OR = 2.03, 95% CI [1.5–2.7], *P* = 4.2e-4; “pronominal reversal—ever”, OR = 2.01, 95% CI [1.5–2.7], *P* = 3.6e-4; “stereotypic use of objects or interest in parts of objects—ever”, OR = 1.92, 95% CI [1.4–2.6], *P* = 2.4e-3; “never nods at age 4–5”, OR = 1.82, 95% CI [1.3–2.5], *P* = 4.9e-3; “little use of pointing to express interest at age 4–5”, OR = 1.80, 95% CI [1.3–2.4], *P* = 4.2e-3; and “limited comprehension of simple language at age 4–5”, OR = 1.73, 95% CI [1.3–2.3], *P* = 1.4e-2. When communication atypicalities requiring a verbal level were excluded from the full sample, the same selected atypicalities as those found for verbal individuals were still highly significantly associated with ER (pronominal reversal being excluded) and maintained equivalent effect sizes (OR: 1.68–2.04). Ten other atypicalities reached significance in the full sample (Fig. 1 and Supplementary Table S1): “rarely responds to the approaches of other children at age 4 to 5”, OR = 1.86, 95% CI [1.5–2.3], *P* = 5.3e-6; “little attention to voice at age 4–5”, OR = 1.72, 95% CI [1.4–2.2], *P* = 1.7e-4; “generally no reciprocal smiling at age 4–5”, OR = 1.71, 95% CI [1.4–2.1], *P* = 1.4e-4; “little or inappropriate social responses at age 4–5”, OR = 1.65, 95% CI [1.3–2.1], *P* = 1.8e-3; “rarely shows social overture at age 4–5”, OR = 1.60, 95% CI [1.3–2.0], *P* = 1.6e-3; “limited use of instrumental gesture at age 4–5”, OR = 1.51, 95% CI [1.2–1.9], *P* = 3.1e-2; “unusual sensory interest – ever”, OR = 1.51, 95% CI [1.2–1.9], *P* = 2.3e-2; “no attempts to share enjoyment at age 4 to 5”, OR = 1.51, 95% CI [1.2–1.9], *P* = 1.6e-2; “hand and finger mannerisms—ever”, OR = 1.49, 95% CI [1.2–1.9], *P* = 2.4e-2; and “inappropriate facial expression—ever”, OR = 1.46, 95% CI [1.2–1.8], *P* = 4.1e-2. Adjusted *P* values by Bonferroni correction are shown. All analyses were controlled for age at enrollment, nonverbal IQ, and sex.

### Combination of atypicalities associated with early regression

The stepwise analysis, performed with the full set of atypicalities, identified a multivariate model with a significant combination of four atypicalities best associated with a history of ER: “hand leading—ever”, OR = 1.79, 95% CI [1.3–2.6], *P* = 1.3e-3; “pronominal reversal—ever”, OR = 1.72, 95% CI [1.7–2.3], *P* = 6.7e-4; “never shakes head at age 4–5”, OR = 1.75, 95% CI [1.3–2.4], *P* = 6.7e-4; and “stereotypic use of objects or interest in parts of objects—ever”, OR = 1.64, 95% CI [1.2–2.3], *P* = 2.3e-3. The presented values were corrected for age, sex, and nonverbal IQ. These are the four atypicalities most strongly associated with a history of ER and among the five atypicalities with the most significant level of association in verbal individuals (Fig. 1).

The bootstrap analysis, which consists of 1000 iterations of the stepwise analysis in samples created by a random replacement procedure, showed consistency. The four atypicalities most frequently selected by the stepwise analysis in the initial sample were all selected in more than 50% of the iterations and were always significant (Supplementary Table S2).

Autistic children who presented a combination of at least three of the identified atypicalities in the cluster had 5.7-fold greater odds (95% CI [3.4–9.6]; *P* = 1.3e-11) of having experienced ER than children who did not have any. The combination of at least two of these atypicalities increased the odds of having experienced ER by 2.5 times (95% CI [1.9–3.4]; *P* = 1.5e-09) compared with children with one or none.

### Association between the combination of atypicalities and autistic phenotype

**Fig. 2:**
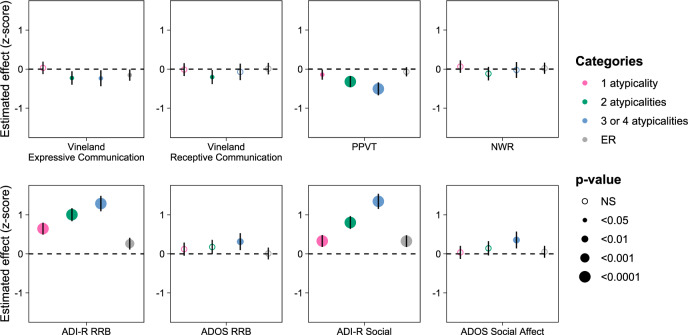
Significant effects of combinations of atypicalities and ER on the areas of communication, language, and severity of symptoms. Standardization of outcome measures allows visual appreciation of effect size. The four atypicalities are as follows: “hand leading—ever”, “pronominal reversal—ever”, “never shakes head at age 4–5”, and “stereotypic use of objects or interest in parts of objects—ever”.

The multiple combinations of the four selected atypicalities were evaluated for their effect on the main autistic area severity proxies. Atypicality combinations were treated as a categorical variable: absence of atypicalities (*n* = 238), presence of one atypicality (*n* = 379), presence of two atypicalities (*n* = 268), presence of three or four atypicalities (*n* = 152). Combinations of three or four atypicalities were grouped in the same category, as very few participants experienced all four atypicalities. The combinations of atypicalities showed little or no association with communicative abilities when measured by the Vineland (Fig. 2 and Supplementary Table S3). Vocabulary knowledge measured by the PPVT showed increasing difficulties with the number of atypicalities (Fig. 2 and Supplementary Table S3). This was not the case for phonological memory measured on the nonword repetition task (Fig. 2 and Supplementary Table S3). The intensity of the autistic phenotype in the dimensions of repetitive and restricted behaviors (RRB), as well as social behaviors, was more severe when the atypicalities were combined or all presented in the individual. This was true when retrospectively assessed with the ADI-R sum scores and at enrollment when measured with the ADOS-calibrated severity scores (Fig. 2 and Supplementary Table S3). ER alone was a poorer predictor than the combination of atypicalities for all outcomes of interest (Fig. 2 and Supplementary Table S3). These analyses were restricted to the sample of verbal participants, as “pronominal reversal” is part of the atypicality cluster and could not be assessed for nonverbal participants.

Participants with a combination of two or more of the atypicalities had significatively greater odds of having received a clinical diagnosis of autistic disorder rather than another diagnosis of the spectrum (based on DSM-IV PDD diagnoses) than participants without any after controlling for nonverbal IQ, sex, and age (one atypicality, OR = 1.25, 95% CI [0.90–1.8], *P* = 1.9e-1; two atypicalities, OR = 2.96, 95% CI [2.0–4.3], *P* = 1.2e-8; three or four atypicalities, OR = 5.7, 95% CI [3.5–9.4], *P* = 4.7e-12). Showing two or more of the atypicalities increased the odds by 3.2-fold (95% CI [2.4–4.2], *P* = 1.6e-16) of having a clinical diagnosis of “autistic disorder” over another PDD diagnosis versus individuals with only one or none of these atypicalities after controlling for nonverbal IQ, sex, and age.

## Discussion

After experiencing ER, autistic children display a developmental plateau [38, 39] that generally resolves into a late recovery of communicative language. Certain atypicalities are overrepresented in individuals for whom ER is reported by caregivers, mainly in communication atypicalities (“pronominal reversal—ever”, “never shakes head at age 4–5”, “hand leading–ever”, “little use of pointing to express interest at age 4–5”, “never nods at age 4–5”, and “limited comprehension of simple language at age 4–5”), as well as perception-based behaviors (“stereotypic use of objects or interest in parts of objects—ever”). Other atypicalities of the communicative and social areas were also overrepresented when the verbal level of the individuals was not considered, the most significant of which were “little attention to voice at age 4–5”, “generally no reciprocal smiling at age 4–5”, and “rarely responds to the approaches of other children at age 4–5”. ER thus initiates an atypical developmental sequence [12, 38–40] characterized by certain atypicalities to a greater extent than that observed in nonregressive individuals. Four of these atypicalities (“hand leading—ever”, “pronominal reversal—ever”, “never shakes head at age 4–5”, “stereotypic use of objects or interest in parts of objects—ever”) tended to occur concomitantly more frequently among individuals with ER than among nonregressive individuals.

The combination of three or more of these four atypicalities was over five times more likely to be found among autistic children who had experienced an ER. The multiple combinations of these four atypicalities, which are mostly in the domain of communication, were associated with a more pronounced autistic phenotype, both in terms of social skills and in terms of repetitive behaviors and restricted interests retrospectively and at the time of assessment. This cluster of atypicalities was not associated with worse phonological memory deficits on the nonword repetition task, although they tended to be associated with more limited vocabulary knowledge. Phonological memory is usually preserved in autistic children [41]. These deficits are instead found mainly in nonautistic neurodevelopmental disorders [32, 42].

Critically, the combination of these atypicalities increased the odds of a clinical diagnosis of autistic disorder rather than another diagnosis on the PDD spectrum. The combination of a minimum of two of these atypicalities, as opposed to the single manifestation or absence of one of these four atypicalities, increased the odds of obtaining a diagnosis of autistic disorder by a factor of three. This group of atypicalities, mostly retrospectively reported in the period of 4–5 years of age, therefore cluster with an overall phenotypic presentation that remains frank at the age of enrollment in our sample (6–18 years). Overall, the copresence of at least two atypicalities in this cluster of four atypicalities constitutes a semiological pattern, which is confirmed by the ADI and ADOS scores and the clinician’s expertise in assessing the overall phenotypic presentation later in development.

### Clinical importance of reported autistic early regression

Overall, the aggregation and contingency of the selected atypicalities, even transient, show their semiological importance in the developmental context that accompanies ER, despite their actual equivalent value in the current system of polythetic criteria. Approximately 40% of the verbal autistic children in our sample shared at least two of the four selected atypicalities.

The strategy used in this study emphasizes the interdependence of a developmental trajectory, usually marked by ER, and the transient presentation of cross-sectional signs. Certain behavioral manifestations may emerge around the same period as the identification of ER, such as the atypical use of objects [17, 39, 43], which is strongly associated with regression in our study. Other atypicalities emerge later in development, accentuating the oddity of language and communicative development without affecting the final adaptive outcome. By granting a “specifier” status to “language impairment” and ignoring ER in the DSM 5 criteria, the current diagnostic formulation underscores the semiological and nosological value of the codependence between the developmental period and manifestation of signs and may undermine the validity of the diagnostic construct [44]. Better integration of developmental trajectories with autistic atypicalities could therefore address the increasing heterogeneity and phenotypic ambiguity of the current criteria [45].

### Limitations

The sample on which this research was conducted is likely to underrepresent children with autism with an intellectual disability. Autistic children with a low nonverbal IQ are less likely to become verbal [46], which amplifies the underrepresentation of these children in some of the analyses of this study.

This is a cross-sectional study, which assumes an equivalent value for the retrospective data used to characterize the participants. However, the time period between the event and its recall is likely to influence the reliability of the measure [8, 47]. Knowledge about the diagnosis is able to influence recall, with a well-informed parent reporting more difficulties in their child’s development [48]. This bias is likely to amplify the observed combination of ER-associated atypicalities but does not invalidate the association found between the copresence of signs.

The choice of using the ADI-R criteria as a definition of ER is questionable. This study does not address the phenotypic distinction that could be associated with different types of regression. Information about ER is obtained retrospectively, which, although not very sensitive, is conservative.

The use of a stepwise analysis to identify the atypicalities best associated with ER is a data-driven type of analysis. As such, the results are dependent on the sample used, and the strength of the associations and their validity may be influenced by selection bias. The SSC participants could be imperfectly representative of the general autistic population since this is not a population-based sample and individuals are issued from simplex families [49]. In addition, each participant had to undergo multiple assessments, which may not be suitable for individuals with low functioning or severe ID, making this subgroup underrepresented in the SSC. This study was limited to a single cohort; replication in another cohort would add validity to the selected group of atypicalities. The bootstrap sensitivity analysis is reassuring with respect to the consistency of the variable selection.

The heterogeneity of individuals included in the autism spectrum as currently defined may seem overinclusive or abstract in clinic and research. The integration of a relatively specific longitudinal dimension, such as a regression or plateau, to the diagnostic criteria, could represent a way to isolate a homogeneous group manifesting key atypicalities within the current autism spectrum to which more heterogeneous individuals can be secondarily compared. Based on the recent argument of the critical role of ER in autism, we used stepwise regression to identify four atypicalities, that when present, are associated with both a higher severity of the autistic areas and the categorical evidence of the diagnosis. Thus, this retrospective study represents a first step in identifying the combination of atypicalities associated with the regressive phenomenon. This method, if applied to the whole inventory of autistic signs, could contribute to limiting the continuous increase in the heterogeneity of the clinical pictures accepted within the spectrum.

## Supplementary information

Figure S1

Table S1

Table S2

Table S3

Appendix S1
