# Supplementary material for: Using developmental regression to reorganize the clinical importance of autistic atypicalities
Source: Transl Psychiatry. 2022 Dec 1;12:498. doi: 10.1038/s41398-022-02263-8 (PMC9715666; doi:10.1038/s41398-022-02263-8)
Supplement: Supplementary file 1 — Figure S1 [file 41398_2022_2263_MOESM1_ESM.docx]

**
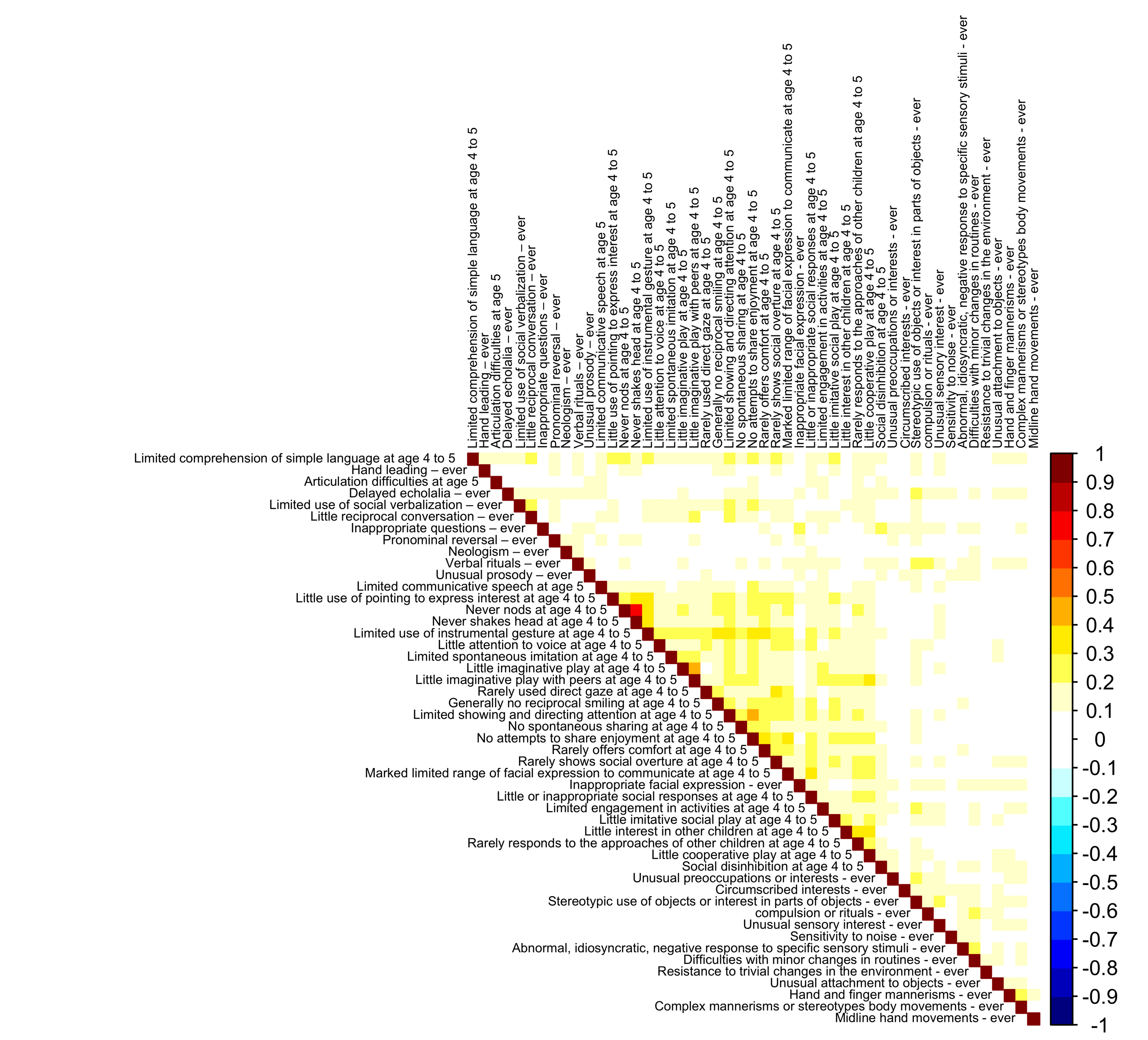
**

**Figure S1**. φ coefficients between atypicalities. The general associations between independent atypicalities are weak in the sample. Only "Never nods" and "Never shakes head" show a phi coefficient of 0.75.
