# Supplementary material for: Using developmental regression to reorganize the clinical importance of autistic atypicalities
Source: Transl Psychiatry. 2022 Dec 1;12:498. doi: 10.1038/s41398-022-02263-8 (PMC9715666; doi:10.1038/s41398-022-02263-8)
Supplement: Supplementary file 2 — Table S1 [file 41398_2022_2263_MOESM2_ESM.docx]

**Table S1.** Coefficients of logistic regressions assessing the association between atypicalities and covariates with ER.

| **Atypicalities** | **ADI-R item** | **atypicality coefficient** | **p-value*** | **Age coefficient** | **p-value** | **nviq coefficient** | **p-value** | **sex coefficient** | **p-value** |
| --- | --- | --- | --- | --- | --- | --- | --- | --- | --- |
| **Verbal individuals** |  |  |  |  |  |  |  |  |  |
| Limited comprehension of simple language at age 4 to 5 | q29_at_4_5 | 5.5E-01 | 2.9E-04 | 4.7E-03 | 1.5E-02 | -8.7E-03 | 3.1E-02 | 2.2E-01 | 3.7E-01 |
| Hand leading – ever | q31_ever | 7.5E-01 | 1.7E-05 | 4.6E-03 | 1.6E-02 | -1.1E-02 | 7.8E-03 | 2.4E-01 | 3.2E-01 |
| Articulation difficulties at age 5 | q32_at_5 | 7.0E-02 | 6.9E-01 | 4.2E-03 | 2.8E-02 | -1.1E-02 | 4.5E-03 | 2.3E-01 | 3.6E-01 |
| Delayed echolalia – ever | q33_ever | 2.7E-01 | 9.6E-02 | 4.3E-03 | 2.4E-02 | -1.1E-02 | 7.3E-03 | 2.3E-01 | 3.5E-01 |
| Limited use of social verbalization – ever | q34_ever | 9.2E-02 | 6.0E-01 | 4.2E-03 | 2.9E-02 | -1.1E-02 | 3.8E-03 | 2.2E-01 | 3.6E-01 |
| Little reciprocal conversation – ever | q35_ever | 3.3E-01 | 1.2E-01 | 4.2E-03 | 2.7E-02 | -1.1E-02 | 6.8E-03 | 2.4E-01 | 3.2E-01 |
| Inappropriate questions – ever | q36_ever | 3.7E-02 | 8.0E-01 | 4.2E-03 | 2.8E-02 | -1.2E-02 | 3.4E-03 | 2.3E-01 | 3.5E-01 |
| Pronominal reversal – ever | q37_ever | 7.0E-01 | 7.3E-06 | 5.3E-03 | 6.7E-03 | -9.8E-03 | 1.4E-02 | 2.5E-01 | 3.1E-01 |
| Neologism – ever | q38_ever | 3.3E-01 | 1.2E-01 | 4.2E-03 | 2.6E-02 | -1.2E-02 | 3.2E-03 | 2.3E-01 | 3.5E-01 |
| Verbal rituals – ever | q39_ever | 5.2E-01 | 1.4E-03 | 4.6E-03 | 1.6E-02 | -9.7E-03 | 1.4E-02 | 2.2E-01 | 3.7E-01 |
| Unusual prosody – ever | q40_ever | 3.7E-01 | 1.3E-02 | 3.8E-03 | 4.7E-02 | -1.2E-02 | 2.6E-03 | 2.4E-01 | 3.4E-01 |
| Limited communicative speech at age 5 | q41_at_5 | 3.7E-01 | 6.6E-02 | 4.0E-03 | 4.0E-02 | -1.1E-02 | 6.7E-03 | 2.3E-01 | 3.5E-01 |
| Little use of pointing to express interest at age 4 to 5 | q42_at_4_5 | 5.9E-01 | 8.6E-05 | 3.6E-03 | 6.0E-02 | -1.2E-02 | 3.3E-03 | 2.7E-01 | 2.8E-01 |
| Never nods at age 4 to 5 | q43_at_4_5 | 6.0E-01 | 1.0E-04 | 4.2E-03 | 3.0E-02 | -1.0E-02 | 1.1E-02 | 2.1E-01 | 3.9E-01 |
| Never shakes head at age 4 to 5 | q44_at_4_5 | 7.1E-01 | 8.5E-06 | 4.1E-03 | 3.2E-02 | -1.0E-02 | 7.9E-03 | 2.5E-01 | 3.2E-01 |
| Limited use of instrumental gesture at age 4 to 5 | q45_at_4_5 | 3.4E-01 | 2.3E-02 | 3.9E-03 | 4.3E-02 | -1.1E-02 | 3.5E-03 | 2.0E-01 | 4.1E-01 |
| Little attention to voice at age 4 to 5 | q46_at_4_5 | 5.1E-01 | 1.1E-03 | 4.0E-03 | 3.9E-02 | -1.3E-02 | 1.4E-03 | 1.8E-01 | 4.6E-01 |
| Limited spontaneous imitation at age 4 to 5 | q47_at_4_5 | 4.0E-01 | 4.0E-02 | 4.2E-03 | 2.9E-02 | -1.2E-02 | 3.0E-03 | 1.9E-01 | 4.5E-01 |
| Little imaginative play at age 4 to 5 | q48_at_4_5 | 2.7E-01 | 1.1E-01 | 4.2E-03 | 2.7E-02 | -1.1E-02 | 4.8E-03 | 1.9E-01 | 4.3E-01 |
| Little imaginative play with peers at age 4 to 5 | q49_at_4_5 | 7.7E-02 | 6.6E-01 | 4.2E-03 | 2.8E-02 | -1.1E-02 | 4.0E-03 | 2.2E-01 | 3.7E-01 |
| Rarely used direct gaze at age 4 to 5 | q50_at_4_5 | -1.8E-02 | 9.0E-01 | 4.2E-03 | 2.7E-02 | -1.1E-02 | 3.4E-03 | 2.3E-01 | 3.6E-01 |
| Generally. no reciprocal smiling at age 4 to 5 | q51_at_4_5 | 4.8E-01 | 1.4E-03 | 3.8E-03 | 4.8E-02 | -1.2E-02 | 2.5E-03 | 2.4E-01 | 3.4E-01 |
| Limited showing and directing attention at age 4 to 5 | q52_at_4_5 | 7.6E-02 | 6.2E-01 | 4.1E-03 | 3.3E-02 | -1.1E-02 | 4.2E-03 | 2.2E-01 | 3.6E-01 |
| No spontaneous sharing at age 4 to 5 | q53_at_4_5 | -1.5E-02 | 9.3E-01 | 4.2E-03 | 2.7E-02 | -1.2E-02 | 3.3E-03 | 2.3E-01 | 3.6E-01 |
| No attempts to share enjoyment at age 4 to 5 | q54_at_4_5 | 3.1E-01 | 4.0E-02 | 3.8E-03 | 4.6E-02 | -1.1E-02 | 6.1E-03 | 2.3E-01 | 3.5E-01 |
| Rarely offers comfort at age 4 to 5 | q55_at_4_5 | 2.2E-01 | 1.5E-01 | 4.0E-03 | 3.7E-02 | -1.1E-02 | 3.6E-03 | 2.2E-01 | 3.8E-01 |
| Rarely shows social overture at age 4 to 5 | q56_at_4_5 | 4.0E-01 | 7.4E-03 | 3.9E-03 | 4.1E-02 | -1.1E-02 | 5.6E-03 | 2.3E-01 | 3.5E-01 |
| Marked limited range of facial expression to communicate at age 4 to 5 | q57_at_4_5 | 2.3E-01 | 1.3E-01 | 4.0E-03 | 3.7E-02 | -1.1E-02 | 4.9E-03 | 2.2E-01 | 3.7E-01 |
| Inappropriate facial expression - ever | q58_ever | 3.9E-01 | 1.1E-02 | 4.2E-03 | 2.9E-02 | -1.1E-02 | 5.5E-03 | 2.3E-01 | 3.6E-01 |
| Little or inappropriate social responses at age 4 to 5 | q59_at_4_5 | 3.6E-01 | 2.0E-02 | 3.8E-03 | 5.2E-02 | -1.1E-02 | 4.6E-03 | 2.2E-01 | 3.6E-01 |
| Limited engagement in activities at age 4 to 5 | q60_at_4_5 | 2.3E-01 | 1.3E-01 | 4.2E-03 | 2.9E-02 | -1.1E-02 | 8.3E-03 | 2.1E-01 | 3.9E-01 |
| Little imitative social play at age 4 to 5 | q61_at_4_5 | -1.1E-02 | 9.4E-01 | 4.2E-03 | 2.7E-02 | -1.2E-02 | 3.3E-03 | 2.3E-01 | 3.6E-01 |
| Little interest in other children at age 4 to 5 | q62_at_4_5 | 2.4E-01 | 1.1E-01 | 3.7E-03 | 5.9E-02 | -1.2E-02 | 2.7E-03 | 2.1E-01 | 4.0E-01 |
| Rarely responds to the approaches of other children at age 4 to 5 | q63_at_4_5 | 4.6E-01 | 3.7E-03 | 3.9E-03 | 4.2E-02 | -1.1E-02 | 7.6E-03 | 1.9E-01 | 4.3E-01 |
| Little cooperative play at age 4 to 5 | q64_at_4_5 | 6.7E-02 | 6.9E-01 | 4.2E-03 | 2.8E-02 | -1.1E-02 | 4.0E-03 | 2.2E-01 | 3.6E-01 |
| Social disinhibition at age 4 to 5 | q66_at_4_5 | -1.3E-01 | 4.0E-01 | 4.1E-03 | 3.1E-02 | -1.2E-02 | 2.7E-03 | 2.2E-01 | 3.6E-01 |
| Unusual preoccupations or interests - ever | q67_ever | 3.5E-01 | 4.7E-02 | 4.4E-03 | 2.1E-02 | -1.1E-02 | 6.4E-03 | 1.8E-01 | 4.8E-01 |
| Circumscribed interests - ever | q68_ever | 1.5E-01 | 3.7E-01 | 4.0E-03 | 3.7E-02 | -1.2E-02 | 3.2E-03 | 2.1E-01 | 4.0E-01 |
| Stereotypic use of objects or interest in parts of objects - ever | q69_ever | 6.5E-01 | 4.8E-05 | 5.0E-03 | 9.1E-03 | -1.0E-02 | 1.2E-02 | 1.4E-01 | 5.6E-01 |
| compulsion or rituals - ever | q70_ever | 3.4E-01 | 2.4E-02 | 4.1E-03 | 3.4E-02 | -1.2E-02 | 3.0E-03 | 2.2E-01 | 3.6E-01 |
| Unusual sensory interest - ever | q71_ever | 3.6E-01 | 2.9E-02 | 4.5E-03 | 2.0E-02 | -1.0E-02 | 8.1E-03 | 2.3E-01 | 3.6E-01 |
| Sensitivity to noise - ever | q72_ever | 5.2E-01 | 2.5E-03 | 4.2E-03 | 2.7E-02 | -1.2E-02 | 2.3E-03 | 2.3E-01 | 3.4E-01 |
| Abnormal, idiosyncratic, negative response to specific sensory stimuli - ever | q73_ever | 2.7E-01 | 6.8E-02 | 4.0E-03 | 3.7E-02 | -1.2E-02 | 3.2E-03 | 2.3E-01 | 3.6E-01 |
| Difficulties with minor changes in routines - ever | q74_ever | 3.6E-01 | 2.0E-02 | 3.9E-03 | 4.4E-02 | -1.2E-02 | 2.2E-03 | 2.3E-01 | 3.4E-01 |
| Resistance to trivial changes in the environment - ever | q75_ever | 3.9E-01 | 7.9E-02 | 4.3E-03 | 2.5E-02 | -1.1E-02 | 5.0E-03 | 2.4E-01 | 3.3E-01 |
| Unusual attachment to objects - ever | q76_ever | 3.4E-01 | 5.8E-02 | 4.3E-03 | 2.5E-02 | -1.1E-02 | 4.2E-03 | 2.2E-01 | 3.6E-01 |
| Hand and finger mannerisms - ever | q77_ever | 3.0E-01 | 4.3E-02 | 4.4E-03 | 2.2E-02 | -1.1E-02 | 7.9E-03 | 2.1E-01 | 3.9E-01 |
| Complex mannerisms or stereotypes body movements - ever | q78_ever | 2.8E-01 | 6.8E-02 | 4.5E-03 | 1.9E-02 | -1.1E-02 | 5.5E-03 | 2.2E-01 | 3.6E-01 |
| Midline hand movements - ever | q79_ever | 4.7E-01 | 2.6E-01 | 4.2E-03 | 2.8E-02 | -1.1E-02 | 3.8E-03 | 2.4E-01 | 3.3E-01 |
| **Full sample** |  |  |  |  |  |  |  |  |  |
| Limited comprehension of simple language at age 4 to 5 | q29_at_4_5 | 2.8E-05 | 8.3E-04 | 5.8E-01 | -1.7E-02 | 3.5E-13 | 2.1E-01 | 2.2E-01 | 2.8E-05 |
| Hand leading – ever | q31_ever | 2.1E-08 | 9.8E-04 | 5.1E-01 | -1.7E-02 | 3.1E-14 | 2.3E-01 | 1.9E-01 | 2.1E-08 |
| Little use of pointing to express interest at age 4 to 5 | q42_at_4_5 | 9.9E-08 | -3.4E-05 | 9.8E-01 | -1.8E-02 | 7.9E-16 | 2.2E-01 | 2.0E-01 | 9.9E-08 |
| Never nods at age 4 to 5 | q43_at_4_5 | 5.1E-09 | 1.0E-03 | 5.0E-01 | -1.5E-02 | 6.5E-11 | 2.1E-01 | 2.2E-01 | 5.1E-09 |
| Never shakes head at age 4 to 5 | q44_at_4_5 | 6.9E-07 | 6.0E-04 | 6.9E-01 | -1.6E-02 | 3.0E-13 | 2.3E-01 | 1.8E-01 | 6.9E-07 |
| Limited use of instrumental gesture at age 4 to 5 | q45_at_4_5 | 7.9E-04 | 3.1E-04 | 8.3E-01 | -1.8E-02 | 6.7E-17 | 1.9E-01 | 2.8E-01 | 7.9E-04 |
| Little attention to voice at age 4 to 5 | q46_at_4_5 | 4.4E-06 | 2.2E-04 | 8.8E-01 | -2.0E-02 | 1.7E-20 | 1.7E-01 | 3.4E-01 | 4.4E-06 |
| Limited spontaneous imitation at age 4 to 5 | q47_at_4_5 | 2.8E-03 | 4.0E-04 | 7.9E-01 | -1.9E-02 | 4.6E-19 | 1.6E-01 | 3.5E-01 | 2.8E-03 |
| Little imaginative play at age 4 to 5 | q48_at_4_5 | 3.9E-02 | 5.7E-04 | 7.0E-01 | -1.9E-02 | 7.6E-18 | 1.8E-01 | 3.1E-01 | 3.9E-02 |
| Little imaginative play with peers at age 4 to 5 | q49_at_4_5 | 2.9E-02 | 5.2E-04 | 7.2E-01 | -1.9E-02 | 1.4E-17 | 1.9E-01 | 2.7E-01 | 2.9E-02 |
| Rarely used direct gaze at age 4 to 5 | q50_at_4_5 | 3.7E-01 | 3.7E-04 | 8.1E-01 | -2.0E-02 | 1.7E-20 | 2.0E-01 | 2.4E-01 | 3.7E-01 |
| Generally. no reciprocal smiling at age 4 to 5 | q51_at_4_5 | 3.5E-06 | 1.4E-06 | 1.0E+00 | -1.9E-02 | 8.9E-19 | 1.9E-01 | 2.7E-01 | 3.5E-06 |
| Limited showing and directing attention at age 4 to 5 | q52_at_4_5 | 7.7E-03 | 1.3E-04 | 9.3E-01 | -1.8E-02 | 3.8E-16 | 1.9E-01 | 2.6E-01 | 7.7E-03 |
| No spontaneous sharing at age 4 to 5 | q53_at_4_5 | 1.8E-01 | 4.1E-04 | 7.8E-01 | -1.9E-02 | 3.7E-19 | 2.0E-01 | 2.5E-01 | 1.8E-01 |
| No attempts to share enjoyment at age 4 to 5 | q54_at_4_5 | 4.0E-04 | -6.8E-05 | 9.6E-01 | -1.8E-02 | 5.1E-17 | 2.1E-01 | 2.3E-01 | 4.0E-04 |
| Rarely offers comfort at age 4 to 5 | q55_at_4_5 | 4.6E-03 | 2.5E-04 | 8.7E-01 | -1.9E-02 | 2.2E-17 | 1.9E-01 | 2.7E-01 | 4.6E-03 |
| Rarely shows social overture at age 4 to 5 | q56_at_4_5 | 4.1E-05 | 1.1E-04 | 9.4E-01 | -1.9E-02 | 7.9E-18 | 2.1E-01 | 2.2E-01 | 4.1E-05 |
| Marked limited range of facial expression to communicate at age 4 to 5 | q57_at_4_5 | 8.0E-03 | 1.7E-06 | 1.0E+00 | -1.9E-02 | 1.2E-18 | 2.1E-01 | 2.2E-01 | 8.0E-03 |
| Inappropriate facial expression - ever | q58_ever | 1.0E-03 | 3.5E-04 | 8.1E-01 | -1.9E-02 | 1.4E-18 | 2.0E-01 | 2.6E-01 | 1.0E-03 |
| Little or inappropriate social responses at age 4 to 5 | q59_at_4_5 | 4.7E-05 | -1.1E-05 | 9.9E-01 | -1.8E-02 | 1.6E-17 | 1.8E-01 | 3.0E-01 | 4.7E-05 |
| Limited engagement in activities at age 4 to 5 | q60_at_4_5 | 5.7E-03 | 3.8E-04 | 8.0E-01 | -1.8E-02 | 3.2E-16 | 1.7E-01 | 3.1E-01 | 5.7E-03 |
| Little imitative social play at age 4 to 5 | q61_at_4_5 | 3.0E-02 | 4.6E-04 | 7.6E-01 | -1.9E-02 | 1.8E-18 | 1.9E-01 | 2.8E-01 | 3.0E-02 |
| Little interest in other children at age 4 to 5 | q62_at_4_5 | 3.7E-03 | -7.7E-05 | 9.6E-01 | -1.9E-02 | 1.8E-18 | 1.6E-01 | 3.4E-01 | 3.7E-03 |
| Rarely responds to the approaches of other children at age 4 to 5 | q63_at_4_5 | 1.4E-07 | 4.7E-04 | 7.6E-01 | -1.7E-02 | 1.5E-14 | 1.7E-01 | 3.2E-01 | 1.4E-07 |
| Little cooperative play at age 4 to 5 | q64_at_4_5 | 1.1E-01 | 5.1E-04 | 7.3E-01 | -1.9E-02 | 3.0E-18 | 2.0E-01 | 2.4E-01 | 1.1E-01 |
| Social disinhibition at age 4 to 5 | q66_at_4_5 | 4.4E-01 | 4.7E-04 | 7.5E-01 | -2.0E-02 | 4.1E-20 | 2.0E-01 | 2.5E-01 | 4.4E-01 |
| Unusual preoccupations or interests - ever | q67_ever | 1.2E-01 | 6.0E-04 | 6.9E-01 | -2.0E-02 | 1.0E-19 | 1.8E-01 | 3.1E-01 | 1.2E-01 |
| Circumscribed interests - ever | q68_ever | 1.6E-01 | 3.1E-04 | 8.4E-01 | -2.0E-02 | 8.8E-21 | 2.0E-01 | 2.6E-01 | 1.6E-01 |
| Stereotypic use of objects or interest in parts of objects - ever | q69_ever | 1.1E-05 | 9.4E-04 | 5.3E-01 | -1.8E-02 | 4.0E-17 | 1.7E-01 | 3.3E-01 | 1.1E-05 |
| compulsion or rituals - ever | q70_ever | 2.4E-02 | 2.8E-04 | 8.5E-01 | -2.0E-02 | 3.1E-20 | 2.1E-01 | 2.2E-01 | 2.4E-02 |
| Unusual sensory interest - ever | q71_ever | 5.8E-04 | 7.6E-04 | 6.1E-01 | -1.8E-02 | 6.7E-17 | 2.1E-01 | 2.4E-01 | 5.8E-04 |
| Sensitivity to noise - ever | q72_ever | 2.6E-02 | 4.1E-04 | 7.8E-01 | -2.0E-02 | 5.1E-21 | 1.9E-01 | 2.8E-01 | 2.6E-02 |
| Abnormal, idiosyncratic, negative response to specific sensory stimuli - ever | q73_ever | 1.9E-02 | 3.5E-04 | 8.1E-01 | -2.0E-02 | 1.8E-20 | 2.2E-01 | 2.0E-01 | 1.9E-02 |
| Difficulties with minor changes in routines - ever | q74_ever | 6.0E-02 | 2.2E-04 | 8.8E-01 | -2.0E-02 | 4.8E-21 | 2.1E-01 | 2.3E-01 | 6.0E-02 |
| Resistance to trivial changes in the environment - ever | q75_ever | 1.7E-01 | 5.2E-04 | 7.3E-01 | -2.0E-02 | 2.1E-20 | 2.1E-01 | 2.3E-01 | 1.7E-01 |
| Unusual attachment to objects - ever | q76_ever | 3.5E-01 | 4.9E-04 | 7.4E-01 | -2.0E-02 | 2.4E-20 | 2.0E-01 | 2.5E-01 | 3.5E-01 |
| Hand and finger mannerisms - ever | q77_ever | 6.1E-04 | 6.5E-04 | 6.6E-01 | -1.8E-02 | 2.9E-16 | 1.9E-01 | 2.7E-01 | 6.1E-04 |
| Complex mannerisms or stereotypes body movements - ever | q78_ever | 1.7E-03 | 9.2E-04 | 5.4E-01 | -1.9E-02 | 8.2E-19 | 2.0E-01 | 2.5E-01 | 1.7E-03 |
| Midline hand movements - ever | q79_ever | 3.9E-01 | 4.7E-04 | 7.5E-01 | -2.0E-02 | 1.7E-20 | 2.1E-01 | 2.3E-01 | 3.9E-01 |

*unadjusted p-value are shown
