# Supplementary material for: Using developmental regression to reorganize the clinical importance of autistic atypicalities
Source: Transl Psychiatry. 2022 Dec 1;12:498. doi: 10.1038/s41398-022-02263-8 (PMC9715666; doi:10.1038/s41398-022-02263-8)
Supplement: Supplementary file 3 — Table S2 [file 41398_2022_2263_MOESM3_ESM.docx]

**Table S2**. Frequency of selection. by stepwise analyses. of atypicalities associated with ER among the obtained combination models after 1000 iterations with the bootstrap procedure.

| **Autistic areas atypicalities** | **Selection Frequency**  **(%)** | **Significant when selected**  **(%)** | **Sign estimate** |
| --- | --- | --- | --- |
| Never shakes head at age 4 to 5 | 60.3 | 100 | + |
| Pronominal reversal - ever | 58.7 | 100 | + |
| Hand leading - ever | 56.9 | 100 | + |
| Stereotypic use of objects or interest in parts of objects - ever | 50.2 | 100 | + |
| Sensitivity to noise - ever | 41.3 | 100 | + |
| Little use of pointing to express interest at age 4 to 5 | 39.8 | 100 | + |
| Social disinhibition at age 4 to 5 | 33.5 | 100 | - |
| Little imitative social play at age 4 to 5 | 29.2 | 100 | - |
| Limited comprehension of simple language at age 4 to 5 | 26.9 | 100 | + |
| Generally. no reciprocal smiling at age 4 to 5 | 22.2 | 100 | + |
| Unusual prosody - ever | 20.3 | 100 | + |
| Verbal rituals - ever | 19.8 | 100 | + |
| Rarely responds to the approaches of other children at age 4 to 5 | 19.8 | 100 | + |
| Rarely used direct gaze at age 4 to 5 | 19.6 | 100 | - |
| Little attention to voice at age 4 to 5 | 18.8 | 100 | + |
| Difficulties with minor changes in routines - ever | 15.6 | 100 | + |
| No spontaneous sharing at age 4 to 5 | 12.4 | 100 | - |
| Limited showing and directing attention at age 4 to 5 | 12.0 | 100 | - |
| Inappropriate facial expression - ever | 10.4 | 100 | + |
| Resistance to trivial changes in the environment - ever | 10.4 | 100 | +/- |
| Rarely shows social overture at age 4 to 5 | 9.9 | 100 | + |
| Never nods at age 4 to 5 | 8.3 | 100 | +/- |
| Little interest in other children at age 4 to 5 | 8.2 | 100 | + |
| Little or inappropriate social responses at age 4 to 5 | 8.1 | 100 | + |
| Unusual preoccupations or interests - ever | 8.1 | 100 | + |
| Hand and finger mannerisms - ever | 8.1 | 100 | +/- |
| Little cooperative play at age 4 to 5 | 7.4 | 100 | +/- |
| Little imaginative play with peers at age 4 to 5 | 5.2 | 100 | - |
| Unusual sensory interest - ever | 4.2 | 100 | + |
| Complex mannerisms or stereotypes body movements - ever | 4.1 | 100 | +/- |
| Limited use of social verbalization - ever | 4.0 | 100 | +/- |
| No attempts to share enjoyment at age 4 to 5 | 4.0 | 100 | +/- |
| Inappropriate questions - ever | 3.6 | 100 | +/- |
| Limited spontaneous imitation at age 4 to 5 | 3.2 | 100 | +/- |
| compulsion or rituals - ever | 3.2 | 100 | +/- |
| Abnormal, idiosyncratic, negative response to specific sensory stimuli - ever | 3.1 | 100 | + |
| Circumscribed interests - ever | 2.4 | 100 | +/- |
| Limited communicative speech at age 5 | 2.3 | 100 | + |
| Limited use of instrumental gesture at age 4 to 5 | 1.9 | 100 | +/- |
| Limited engagement in activities at age 4 to 5 | 1.7 | 100 | +/- |
| Unusual attachment to objects - ever | 1.7 | 100 | +/- |
| Midline hand movements - ever | 1.7 | 100 | +/- |
| Articulation difficulties at age 5 | 1.6 | 100 | +/- |
| Delayed echolalia - ever | 1.5 | 100 | +/- |
| Marked limited range of facial expression to communicate at age 4 to 5 | 1.5 | 100 | +/- |
| Little reciprocal conversation - ever | 1.3 | 100 | +/- |
| Rarely offers comfort at age 4 to 5 | 1.3 | 100 | +/- |
| Little imaginative play at age 4 to 5 | 0.9 | 100 | +/- |
| Neologism - ever | 0.5 | 100 | +/- |
