# Supplementary material for: Using developmental regression to reorganize the clinical importance of autistic atypicalities
Source: Transl Psychiatry. 2022 Dec 1;12:498. doi: 10.1038/s41398-022-02263-8 (PMC9715666; doi:10.1038/s41398-022-02263-8)
Supplement: Supplementary file 4 — Table S3 [file 41398_2022_2263_MOESM4_ESM.docx]

**Table S3**. Linear regression coefficients of the association between combinations of atypicalities or ER and covariates with z-scored proxy outcomes of communication, language and severity of autistic areas.

| **Outcome** | **1 atypicality coefficient** | **p-value** | **2 atypicalities coefficient** | **p-value** | **3 atypicalities coefficient** | **p-value** | **NVIQ coefficient** | **p-value** | **Sex coefficient** | **p-value** | **Age coefficient** | **p-value** |
| --- | --- | --- | --- | --- | --- | --- | --- | --- | --- | --- | --- | --- |
| Vineland Expressive communication* | 3,3E-02 | 6,9E-01 | -2,3E-01 | 1,0E-02 | -2,3E-01 | 2,5E-02 | 1,8E-02 | 2,6E-26 | 1,1E-03 | 9,9E-01 |  |  |
| Vineland Receptive communication | -1,1E-02 | 9,0E-01 | -2,0E-01 | 2,6E-02 | -7,0E-02 | 5,1E-01 | 1,3E-02 | 3,4E-15 | -9,3E-02 | 3,5E-01 |  |  |
| PPVT | -1,4E-01 | 3,1E-02 | -3,2E-01 | 8,5E-06 | -5,1E-01 | 2,9E-09 | 3,2E-02 | 1,4E-102 | -1,4E-01 | 7,8E-02 |  |  |
| CTOPP - NWR | 6,4E-02 | 4,3E-01 | -1,2E-01 | 1,8E-01 | 1,7E-02 | 8,7E-01 | 1,9E-02 | 3,6E-31 | -7,5E-02 | 4,0E-01 |  |  |
| ADI-R RRB | 9,1E-02 | 2,1E-01 | 4,6E-01 | 6,8E-08 | 6,3E-01 | 9,5E-09 | -4,3E-03 | 8,2E-03 | 3,2E-01 | 4,5E-04 | -5,4E-04 | 5,0E-01 |
| ADOS RRB | 9,7E-02 | 1,9E-01 | 1,5E-01 | 8,5E-02 | 3,0E-01 | 7,9E-03 | -5,6E-03 | 7,8E-04 | 3,4E-01 | 2,6E-04 |  |  |
| ADI-R Social | 5,7E-01 | 6,3E-17 | 9,1E-01 | 6,3E-30 | 1,4E+00 | 4,0E-38 | 2,0E-04 | 8,9E-01 | 2,0E-01 | 2,0E-02 | 3,0E-03 | 5,4E-05 |
| ADOS Social Affect | -2,6E-02 | 7,2E-01 | 1,5E-01 | 8,8E-02 | 3,8E-01 | 7,0E-04 | -7,0E-03 | 4,3E+00 | 1,1E-01 | 2,5E-01 |  |  |
| **Outcome** | **ER coefficient** | **p-value** |  |  |  |  | **NVIQ coefficient** | **p-value** | **Sex coefficient** | **p-value** | **Age coefficient** | **p-value** |
| Vineland Expressive communication* | -1,6E-01 | 7,9E-02 |  |  |  |  | 1,8E-02 | 8,7E-31 | 5,9E-03 | 9,5E-01 |  |  |
| Vineland Receptive communication | 3,1E-02 | 7,5E-01 |  |  |  |  | 1,4E-02 | 1,6E-17 | -7,0E-02 | 4,5E-01 |  |  |
| PPVT | -2,3E-01 | 3,0E-03 |  |  |  |  | 3,3E-02 | 6,8E-117 | -1,3E-01 | 9,1E-02 |  |  |
| CTOPP- NWR | 2,3E-03 | 9,8E-01 |  |  |  |  | 1,9E-02 | 1,2E-32 | -7,7E-02 | 3,9E-01 |  |  |
| ADI-R RRB | 2,4E-01 | 1,4E-02 |  |  |  |  | -6,4E-03 | 9,0E-05 | 3,2E-01 | 7,0E-04 | -9,6E-04 | 2,4E-01 |
| ADOS RRB | 8,7E-02 | 3,7E-01 |  |  |  |  | -6,4E-03 | 8,9E-05 | 3,4E-01 | 2,9E-04 |  |  |
| ADI-R Social | 3,9E-01 | 5,7E-05 |  |  |  |  | -4,1E-03 | 1,1E-02 | 1,9E-01 | 5,0E-02 | 2,0E-03 | 1,3E-02 |
| ADOS Social Affect | 6,9E-02 | 4,8E-01 |  |  |  |  | -8,1E-03 | 7,5E-07 | 1,1E-01 | 2,3E-01 |  |  |
