# Supplementary material for: Using developmental regression to reorganize the clinical importance of autistic atypicalities
Source: Transl Psychiatry. 2022 Dec 1;12:498. doi: 10.1038/s41398-022-02263-8 (PMC9715666; doi:10.1038/s41398-022-02263-8)
Supplement: Supplementary file 5 — Appendix S1 [file 41398_2022_2263_MOESM5_ESM.docx]

**Appendix S1:**

*List of R packages used for the analyses*

For logistic and linear regressions. we used built-in functions in R version 4.1.3. For other analyses. we used specific packages.

- Stepwise selection with bootstrap procedure: boot.stepAIC() function from the R package “bootStepAIC” (1)
- Forest plot: forestplot() function from the R package “forestplot” (2)
- Eulerr plot: eulerr() function from the R package “eulerr” (3)
- Phi association measure: phi() function from the R package “sjstats” (4)
- Plots: ggplot () function was used to generates graphs. from R package “ggplot2” (5)

**References**

1. Rizopoulos D. bootStepAIC: Bootstrap stepAIC [Internet]. 2022. Available from: https://CRAN.R-project.org/package=bootStepAIC

2. Gordon M, Lumley T. forestplot: Advanced Forest Plot Using “grid” Graphics [Internet]. 2021. Available from: https://CRAN.R-project.org/package=forestplot

3. Larsson J. eulerr: Area-Proportional Euler and Venn Diagrams with Ellipses. [Internet]. 2021. Available from: URL: https://CRAN.R-project.org/package=eulerr

4. Lüdecke D. sjstats: Statistical Functions for Regression Models (Version 0.18.1) [Internet]. 2021. Available from: https://CRAN.R-project.org/package=sjstats

5. Wickham H. ggplot2: Elegant Graphics for Data Analysis [Internet]. Springer-Verlag New York; 2016. Available from: https://ggplot2.tidyverse.org
